# Effects of continuous renal replacement therapy on linezolid pharmacokinetic/pharmacodynamics: a systematic review

**DOI:** 10.1186/s13054-016-1551-7

**Published:** 2016-11-19

**Authors:** Gianluca Villa, Paola Di Maggio, A. Raffaele De Gaudio, Andrea Novelli, Riccardo Antoniotti, Enrico Fiaccadori, Chiara Adembri

**Affiliations:** 1 Department of Health Science, Section of Anesthesiology and Intensive Care, University of Florence, Largo Brambilla 3, Florence, 50134 Italy; 2 Department of Health Sciences, Section of Clinical Pharmacology and Oncology, University of Florence, Viale Pieraccini 18, Florence, 50139 Italy; 3 Acute and Chronic Renal Failure Unit, Department of Clinical and Experimental Medicine, Parma University Medical School, Via Gramsci 14, Parma, 43100 Italy; 4 Department of Anesthesia and Intensive Car, Azienda Ospedaliero-Universitaria Careggi, Largo Brambilla 3, Florence, 50134 Italy

**Keywords:** Acute kidney injury, Adsorption, Clearance, Critical illness, High cut-off membranes, High-flux membranes, Linezolid, Sepsis

## Abstract

**Background:**

Major alterations in linezolid pharmacokinetic/pharmacodynamic (PK/PD) parameters might be expected in critically ill septic patients with acute kidney injury (AKI) who are undergoing continuous renal replacement therapy (CRRT). The present review is aimed at describing extracorporeal removal of linezolid and the main PK-PD parameter changes observed in critically ill septic patients with AKI, who are on CRRT.

**Method:**

Citations published on PubMed up to January 2016 were systematically reviewed according to the preferred reporting items for systematic reviews and meta-analyses (PRISMA) statement. All authors assessed the methodological quality of the studies and consensus was used to ensure studies met inclusion criteria. In-vivo studies in adult patients with AKI treated with linezolid and on CRRT were considered eligible for the analysis only if operational settings of the CRRT machine, membrane type, linezolid blood concentrations and main PK-PD parameters were all clearly reported.

**Results:**

Among 68 potentially relevant articles, only 9 were considered eligible for the analysis. Across these, 53 treatments were identified among the 49 patients included (46 treated with high-flux and 3 with high cut-off membranes). Continuous veno-venous hemofiltration (CVVH) was the most frequent treatment performed amongst the studies. The extracorporeal clearance values of linezolid across the different modalities were 1.2–2.3 L/h for CVVH, 0.9–2.2 L/h for hemodiafiltration and 2.3 L/h for hemodialysis, and large variability in PK/PD parameters was reported. The optimal area under the curve/minimum inhibitory concentration (AUC/MIC) ratio was reached for pathogens with an MIC of 4 mg/L in one study only.

**Conclusions:**

Wide variability in linezolid PK/PD parameters has been observed across critically ill septic patients with AKI treated with CRRT. Particular attention should be paid to linezolid therapy in order to avoid antibiotic failure in these patients. Strategies to improve the effectiveness of this antimicrobial therapy (such as routine use of target drug monitoring, increased posology or extended infusion) should be carefully evaluated, both in clinical and research settings.

## Background

Sepsis is frequently observed in the intensive care unit (ICU), and it is one of the major causes of death among critically ill patients [1–4]. Infections in the ICU are frequently driven by multidrug-resistant strains [5], those sustained by Gram-positive bacteria such as methicillin-resistant Staphylococcus aureus or vancomycin-resistant Enterococci being the most frequently observed [2, 6, 7]. Linezolid has been shown to be efficacious against these micro-organisms and, therefore it is frequently used in ICU patients [7, 8].

The volume of distribution (Vd) of this low molecular weight (337 Da) oxazolidinone is 0.5–0.6 L/kg in adult patients [9]; plasma elimination half-life (t_1/2_) ranges from 3.1 to 4.9 h, and clearance is 6.4 to 14.8 L/h [10]. Linezolid is metabolized by the liver to inactive metabolites excreted with the parent substance by the kidneys [11, 12]. The parameters that best describe the antibiotic activity of linezolid are the time during which the plasma concentration exceeds the minimum inhibitory concentration (MIC) for the specific pathogen (T > MIC), and the area under the plasma concentration time curve over MIC ratio (AUC_0–24_/MIC) [13]. A T > MIC of ≥85 and an AUC_0–24_/MIC >100 are required to exert maximal antimicrobial efficacy, and are associated with better clinical outcome in severely ill patients [13].

It is generally assumed that the recommended linezolid dose of 600 mg every 12 h provides adequate plasma exposure to the antibiotic, and for this reason, along with the safety profile of the molecule, therapeutic drug monitoring (TDM) is deemed unnecessary [14, 15]. However, wide variability in serum antibiotic concentrations has been reported among critically ill patients [2, 16], the concentrations below the therapeutic range being associated with development of antibiotic resistance and failure of antimicrobial therapy [17], while concentrations above this range may increase the risk of adverse reactions [18]. Possible drug-drug interactions may contribute to the variability of linezolid serum concentrations. Organ dysfunction and changes in vascular permeability typically observed among critically ill patients also explain the wide variation in antibiotic serum concentrations reported in the ICU [16, 19]. For example, patients with severe sepsis or septic shock may frequently develop acute kidney injury (AKI) [20], which may affect drug disposition through changes in Vd, protein binding and total body clearance [21, 22]. Nonetheless, it is generally assumed that minimal pharmacokinetic/pharmacodynamic (PK/PD) alterations might be expected for linezolid during septic AKI, as linezolid renal clearance normally accounts for <30% of total clearance and its Vd is minimally affected during sepsis due to the moderate lipophilic nature.

Continuous renal replacement therapy (CRRT), the most widely used modality in patients with sepsis and AKI [23] may further reduce antibiotic blood concentrations by extracorporeal drug removal, increasing the risk of antimicrobial therapy failure [24–26]. Thus, in order to avoid suboptimal exposure, antibiotic therapy may frequently require dose adjustment during CRRT [24].

In patients on antibiotic treatment and CRRT, both specific antibiotic-related and CRRT-related characteristics are involved in extracorporeal drug removal [27]. In the specific case of linezolid, it seems likely that the molecule is efficiently removed by CRRT. As a matter of fact, the molecular weight is 337 Da, 10-fold to 100-fold lower than the cutoff of the “high-flux” or “high cut-off” (HCO) membranes generally used in the ICU, the free fraction of the drug is about 70%, and the Vd is relatively low (0.5–0.6 L/Kg) [13]. Moreover, albumin-bound linezolid can also be removed by HCO membranes (cut-off 60 KDa) [28]. As far as the operational characteristics of CRRT are considered, the elimination of linezolid might be remarkable during both diffusion (due to the low molecular weight) and convection (owing to the characteristics of the most recent high-permeability membranes). Although the non-hydrophilic nature, the transmembrane removal of linezolid during CRRT is directly related to the effluent dose. An effluent dose of 25 ml/Kg/h is nowadays recommended for critically ill patients with AKI, although higher doses may be prescribed for patients with sepsis and AKI (such as during high-volume hemofiltration), potentially leading to an increased removal of antibiotic. Furthermore, because most of the membranes used in the ICU have selective or unselective adsorption properties, small non-hydrophilic molecules such as linezolid may be easily bound and adsorbed from the circulation proportionally to serum concentration.

Thus, both antibiotic-related and CRRT-related characteristics may concomitantly and significantly affect the extracorporeal clearance of linezolid. As a consequence, relevant derangements of serum antibiotic levels are not unexpected in critically ill patients with sepsis and AKI who are undergoing CRRT, and failure to achieve adequate PK-PD targets may lead to ineffective pathogen eradication or increased risk of adverse effects [29].

Thus, this systematic review is aimed at (1) evaluating the effects of CRRT on linezolid removal, with special regard to the different modalities used in the ICU, and (2) describing and discussing the possible CCRT-related factors interfering with achievement of adequate PK-PD targets in critically ill patients with sepsis and AKI.

## Methods

A systematic review was conducted in Pubmed, Embase, Scopus and Web of Science, according to the preferred reporting items for systematic reviews and meta-analyses (PRISMA) statement [30] to describe the main PK-PD parameters observed among critically ill patients with sepsis and AKI who were treated with linezolid and were on CRRT.

The search strategy for the literature selection used was: Linezolid AND (dialysis OR hemofiltration OR hemodiafiltration OR CVVH OR CVVHD OR CVVHDF OR “high cut-off” OR HCO-CVVHD OR “high volume hemofiltration” OR hemadsorption OR adsorption OR AN69 OR AN69ST OR toraymyxin OR cytosorb OR oxiris OR polyacrylonitrile OR polymethylmetacrylate OR polysulfone) AND (PK OR PD OR pharmacokinetic OR pharmacodynamic). The search included citations published up to January 2016; no filter was set on publication dates and language. Studies were eligible for review if they met the following inclusion criteria: (1) included every in-vivo prospective or retrospective study on adult patients with AKI treated with linezolid and CRRT; (2) the flows set in the CRRT machine and the membrane used were clearly indicated; (3) linezolid blood concentrations and main PK-PD parameters were clearly expressed. Studies were excluded if: (1) participants were aged ≤18 years; (2) in-vitro data only were analyzed; (3) intermittent hemodialysis, sustained low-efficiency dialysis or peritoneal dialysis was used.

## Results

**Fig. 1:**
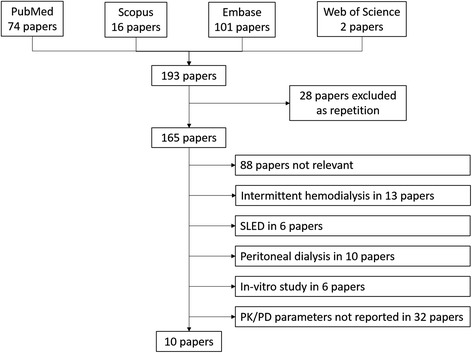
Study selection process. *PK-PD* pharmacokinetics/pharmacodynamics, *SLED* sustained low efficient dialysis

The study selection chart is shown in Fig. 1. The literature search identified 193 potentially relevant articles, which were collected and checked against the eligibility criteria. Only 10 studies met the inclusion criteria and are summarized in Table 1. All studies were based on prospective data, and the papers comprised one congress presentation [31], five small case-series/case reports [9, 24, 32–34], three reports of observational studies [2, 26, 35] and only one report of a randomized clinical trial [36].

**Table 1:** Data on extracorporeal removal and PK/PD parameters obtained from literature analysis

		2016; Roger et al.	2005; Meyer et al.	2005; Meyer et al.	2004; Fiaccadori et al.	2004; Pea et al.	2016. Roger et al.	2012 Carcelero et al.	2006; Mauro et al.	2014; Zoller et al.	2012; Ide et al.	2003; Kraft et al.	2015; Villa et al.	2014; Zoller et al.
		Prospective RCT	Prospective observational study	Prospective observational study	Prospective observational study	Prospective observational case report	Prospective RCT	Prospective observational case report	Prospective observational case report	Prospective observational study	Abstract	Prospective observational case report	Prospective observational case report	Prospective observational study
Treatment parameters	Number of procedures	8	7	13	2	2	9	2	1	2	14	1	3	3
	Treatment	CVVH-post	CVVH-post	CVVH-post	CVVH-pre	CVVH-pre	CVVHDF-post	CVVHDF-pre	CVVHDF-pre	CVVHDF-?	CVVHDF-?	CVVHDF-?	CVVHD	CVVHD
	Membrane	HF	HF	HF	HF	HF	HF	HF	HF	HF	HF	HF	HCO PAES	HF
		PS	PS	PS	PAN	PS	PS	PAN	PAN	PS	PS	PS		PS
		1.2 m^2^	1.2 m^2^	0.9 m^2^	1.65 m^2^	1.25 m^2^	1.2 m^2^	0.9 m^2^	1 m^2^	1.4 m^2^	?	1.6 m^2^	1.1 m^2^	1.8 m^2^
	Qb (ml/min)	200 (185–245)	189 ± 15	185 ± 15	150	125 (120–130)	200 (165–200)	165 (150–180)	200	130 (100–150)	79.3 ± 2.7	200	150	120 (80–150)
	Qd (L/h)						1.14^a^	1 (1–1)	1.2^a^	1.4 (1.2–1.5)	0.52 ± 0.31	2	3^a^ (2.9–3.2)	1.6 (1–2)
	Qf (L/h)	1.67^a^	2.5 ± 0.6	2.3 ± 0.4	2.25 (2–2.5)	2 (2–2)	1.22^a^	1.5 (1–2)	0.2	1.5 (1–2)	0.33 ± 0.15	0.75	-	
	Qf_NET_ (ml/h)	0–200				115 (80–150)	0–200	100 (50–150)		125 (50–200)		774	100	100 (50–200)
	UF_NET_ (L)	3.6 (0.2–4.3)					1.1 (0.4–1.6)							
	BW	76 (55–92)	90 ± 22^a^	83 ± 15^a^	64 (57–72)	64.5 (54–75)	76 (55–92)	67.5 (55–80)	125			100	86 (83–90)	
	Prescribed dose (ml/kg/h)	30			35 (35–35)	33.6 (29–39)	30	41.2^a^ (27–56)	11.2			27.7^a^	30	
	Effective time of treatment (min)	715 (697–751)			675 (630–720)		745 (645–733)			3990				5000
	APACHE II		30 ± 5 (24–40)	26 ± 8 (7–32)	27 (27–27)			27 (26–28)		31.5 (28–35)				25 (23–28)
	SOFA at RRT initiation	12 (10–16)					13 (6–17)	15 (15–15)					14.3 (13–15)	
Parameters of CRRT removal	SA/SC (%)		77 ± 10 (62–86)	69 ± 10 (53–91)	59 (56–61)	84 (76–92)		78 (74–82)			0.86 ± 0.03	79	74 (66–80)	
	Qef (L/h)	1.98^a^	2.5 ± 0.6 (1.5–3)	2.3 ± 0.4 (1.5–3)		2.1 (2.1–2.2)	2.4^a^	2.6^a^ (2.2–3.1)	1.4^a^			2.8	3^a^ (2.9–3.2)	
	Qef (ml/kg/h)	26^a^	27.8^a^ (16.3–40)	27.7^a^ (15–37.5)			32.2^a^						30	
	X_CRRT_ (mg)				89.9 (75–105)	237 (160–314)		218.9 (154–283)	50.1				197.6 (154–266)	
	CL_CRRT_ (L/h)		2.3 ± 0.9 (1.3–4.3)	1.6 ± 0.5 (0.9–2.7)	1.2 (1.2–1.3)	1.4 (1-2–1.6)		2.1 (1.8–2.3)	0.9			2.2	2.3 (2.1–2.5)	
PK and PD parameters	Cmax (mg/L)	~19^b^	12.4 ± 2.3 (7.6–10.5)	16.9 ± 3.8 (11.4–21.9)	19.93 (15.9–23.9)	28.67 (17.1–40.3)	~18^b^	18.85 (16.5 − 21.2)	15.3			16.4	17.13 (10.4–23.5)	
	Cmin (mg/L)	~6^b^	1.7 ± 1.2 (0.3–3.7)	2 ± 1.9 (0.3 − 8)		14.1 (6.5–21.7)	~4^b^	5.4 (5.2–5.6)	3.8	9.4 (4.2–14.5)		7.2	6.2 (2.9–10.3)	8.5 (3.7–18.7)
	T_1/2_ (h)		4.6 ± 1.6 (2.4–7.1)	4.1 ± 1.8 (2.1 − 8.4)	4.6 (2.6–6.5)	15.5 (12.5–-18.5)		6.2 (4.9–7.4)			8.78 ± 3.74	7.5	7.7 (6.1–10.1)	
	AUC_0-∞_ (mg•h/L)	227.9 ± 115	67.6 (34–118)	85.7 (40–244)		444.6 (219–669)	227.9 ± 115	263.5 (214–312)	105.8^a^	303.9 (165–442)	247.9 ± 107.8		208.2 (95–352)	283.1 (144–453)
	Vd (L)	26.5 ± 10.3	60.5 ± 8.6 (42.9–70.8)	46.3 ± 11.1 (29.9–70.7)	31.4 (25.2–37.6)	67.9 (91.5–44.3)	26.5 ± 10.3	44 (29.2–58.8)			31 ± 3.8	49	48.9 (39.9–57.9)	
	CLtot (L/h)	4.5	10.4 ± 3.9 (5.1–17.6)	8.7 ± 3.0 (2.5–14.7)		3.6 (1.8–5.5)	5.9	4.5 (3.6–5.3)	11.3		294 ± 1.38	5.1	3.8 (1.7–6.3)	
	CL_CRRT_/CLtot (%)		22.6^a^	18.7^a^		48.89 (28.7–69.1)		48.9^a^ (33.9–63.9)	7.9^a^			43.1	67.1 (39.6–100)	
	AUC_0-∞_/MIC 4 mg/L		33.8^a^	42.8^a^		111.1 (54–167)		65.9^a^ (53–78)	26.5^a^				52.0 (23–88)	
	AUC_0-∞_/MIC 2 mg/L		67.6^a^	85.7^a^		222.3 (109–334)		131.8^a^ (107–156)	52.9^a^				104.1 (47–176)	
	AUCfree/MIC 4 mg/L												35.9 (16–61)	
	AUCfree/MIC 2 mg/L												71.8 (33–122)	
	% T > MIC 4 mg/L		51 ± 19 (26–88)	61 ± 36 (31–164)										
	% T > MIC 2 mg/L		89 ± 32 (46–137)	96 ± 50 (48–234)										

^a^Calculated from data presented in the original paper. ^b^Derived from figures presented in the original paper. *Qb* blood flow, *Qd* dialysate flow, *Qf* replacement flow, *Qf*_*NET*_ net ultrafiltration flow, *UF*_*NET*_ net ultrafiltrate, *BW* body weight, *APACHE II* Acute Physiology and Chronic Health Evaluation II, *SOFA* Sequential Organ Failure Assessment, *SA/SC* saturation coefficient or sieving coefficient, *Qeff* effluent flow, *X*_*CRRT*_ total amount of drug eliminated by the extracorporeal treatment, *CL*_*CRRT*_ extracorporeal clearance, *Cmax* antibiotic maximum serum concentration, *Cmin* antibiotic trough, *T*_*1/2*_ elimination half-life, *AUC* area under the curve, *Vd* volume of distribution, *CLtot* total clearance

Sixty-seven CRRT treatments were identified across the 10 selected studies; amongst these, 60 were treated with high-flux membranes and 3 with HCO membranes. Continuous veno-venous hemofiltration (CVVH) was the most frequent modality used (28 post-dilution, 4 pre-dilution over 67 treatments 47.8%), with prescribed effluent doses of 30–35 ml/kg/h. On the other hand, 29 treatments over 67 (43.3%) were performed with the continuous veno-venous hemodiafiltration (CVVHDF) modality, and the remaining 6 with continuous veno-venous hemodialysis (CVVHD) (6/67 patients, 8.9%), with a prescribed effluent dose of 30 ml/kg/h for CVVHD and 27.7–41.2 ml/kg/h for CVVHDF. Data from Mauro et al. [34] were excluded because effluent dose values were considered too low [11.2 ml/kg/h]. All but two studies provided information on extracorporeal linezolid removal achieved during CRRT (see Table 1).

## Discussion

Linezolid is a moderately lipophilic drug with limited renal clearance of around 30%. Accordingly, the influence of CRRT in its clearance might be expected to be only moderate. However, wide variability in PK parameters has been reported for linezolid in critically ill patients with sepsis [2, 37], especially when AKI coexists and RRT is needed [36]. This systematic review describes the parameters of extracorporeal removal of linezolid in the course of different modalities of CRRT, and of derangements in PK parameters in critically ill patients with sepsis and AKI, who are on CRRT.

### Effect of dose and modality

Although data considered for this review are only derived from studies of continuous treatments, wide variability in treatment modalities and operational parameters (such as blood, dialysate, replacement flows, etc.) was evident (see Table 1). Despite the wide variability observed, as well as treatment heterogeneity, extracorporeal clearance values for linezolid were similar across the different modalities: 1.2–2.3 L/h for CVVH, 0.9–2.2 L/h for CVVHDF and 2.3 L/h for CVVHD.

Although diffusive techniques should theoretically be characterized by higher extracorporeal clearance for low molecular-weight molecules (like linezolid) when compared with convective techniques, this effect was not observed across the studies assessed. Indeed, a number of factors might have influenced this finding, such as the variability in the flow set of the extracorporeal circuit and/or the specific geometrical characteristics of the various membranes, and the lack of a direct comparison of linezolid removal between the different techniques (diffusive vs convective). In fact, only one study [36] directly compared the PK linezolid parameters in CVVH and CVVHDF. Particularly, this study compared linezolid PK parameters in critically ill patients with sepsis and AKI treated with CVVH or CVVHDF at the same prescribed effluent dose (30 ml/kg/h) [36]. Unfortunately, the authors reported few data specifically for patients on CVVH or CVVHDF. Indeed, excluding the total drug clearance, no other PK parameters were reported or formally compared between the two groups. Furthermore, when comparing the total body clearance, extracorporeal clearance was not detailed (i.e. CVVH or CVVHDF clearance). A 20% reduction in total linezolid clearance (5.9 vs 4.5 L/h, *p* = 0.39) was observed in the group of patients treated with CVVH as compared to CVVHDF, making this finding in line with the concept of higher extracorporeal clearance achievable for small molecules through diffusive techniques. However, although a total effluent dose of 30 ml/kg/h was reported in the method section for both groups of patients, the CVVH group was in fact treated with a 20% lower total effective effluent dose (Qeff) compared to the CVVHDF group (26 ml/kg/h vs 32.17 ml/kg/h) (See Table 1). As the extracorporeal clearance is defined as the sieving coefficient (SC) (or saturation coefficient, SA, for CVVHDF) multiplied by Qeff, it is not surprising that the total linezolid clearance was 20% lower in the CVVH group as compared to CVVHDF (See Table 1). Therefore, in these conditions, it is not possible to infer that removal by diffusive techniques is more efficient than by convective techniques.

### Effect of membrane characteristics

The analysis of the extracorporeal drug removal and PK derangements due to CRRT should also take into account the characteristics of the membrane used, in terms of surface area, composition and pore diameter [38].

Higher extracorporeal clearance of linezolid has been documented in patients treated with larger filters (1.2 vs 0.9 m^2^) [35]. Particularly, although these two groups of patients were treated with the same modality and treatment setting (CVVH in post-dilution, with a blood flow of 185–189 ml/minute and effluent dose of 27.7 ml/kg/h), extracorporeal clearance was higher when the larger filter was used (2.34 L/h vs 1.63 L/h) (see Table 1). Furthermore, membrane composition may also influence drug removal by adsorption. As the most common membranes used in the ICU, such as polymethylmethacrylate or polyacrylonitrile, also have unselective adsorption properties, further studies should take this issue into consideration. In fact, although membrane drug adsorption is commonly underrated, it is usually rapid and not reversible, leading to a reduction of antibiotic concentrations [39]. For example, Kraft et al. [32] and Carcelero et al. [24], using the same treatment modality (CVVHDF) and the same operational setting (effluent flow 2.6–2.7 L/h), observed the same extracorporeal clearance of linezolid (2.05–2.19 L/h), despite the former having used a polysulfone membrane with a twofold surface area (1.6 m^2^) compared to the acrylonitrile membrane used by the latter (0.9 m^2^) (see Table 1). In this case, the reduced transmembrane clearance obtained by the smaller acrylonitrile surface might have been compensated by its higher adsorption properties; as a consequence, the total extracorporeal clearance was similar to that observed with polysulfone membrane with by a broader surface area but lower adsorption capacity.

In the case of linezolid, a molecule with quite a low radius as compared to the large cut-off of the hemodiafilters commonly used in the ICU (high flux and/or HCO membranes), pore size is likely to be scarcely relevant. For example, Villa et al. observed a mean SA equal to 0.80 with HCO membranes used in CVVHD [9]; this value was similar to SA or SC reported in other studies in which patients with AKI were treated with hemodiafiltration or hemofiltration with standard high-flux membranes. In particular, Kraft et al. calculated SA values ranging from 0.77 to 0.81 during CVVHDF [32] and similar results were reported by several authors for the SC obtained during CVVH [24, 33–35]. The removal of linezolid calculated by Villa et al. during HCO-CVVHD was similar to that reported in the literature for high-flux membranes [9], even though an increase in transmembrane linezolid loss should be expected with HCO if convective clearance is applied during the treatment (see aforementioned).

### Effect of patients’ clinical characteristics

Apart from the key role of CRRT in determining PK antibiotic changes, even the critically ill status may be playing a major role [19]. Indeed, increased drug clearance was reported in patients with low SOFA scores [36]. Commonly, in patients with sepsis and AKI, who are undergoing CRRT and are on vasopressor support (as in the study by Roger et al.) [36], lower SOFA scores are mainly correlated with improved neurologic, respiratory and/or liver function. As 40% of the drug is usually metabolized and inactivated by the liver, it is not surprising that lower SOFA scores were associated with higher linezolid clearance.

The results were similar in our previous study performed in patients with sepsis and AKI treated with standard doses of linezolid and hemodialysis with HCO membranes [9], in which impaired liver function was associated with decreased corporeal drug clearance. As the total clearance (CL_tot_) is the sum of the extracorporeal (CL_HCO_) and non CRRT clearance, a subsequent increased ratio between extracorporeal and total linezolid clearance (CL_HCO_/CL_tot_) was observed in critically ill patients with AKI and severe liver dysfunction. In these conditions, the CL_HCO_/CL_tot_ value is likely to be higher than 30%, only apparently identifying a treatment in which the extracorporeal clearance is critical for the total drug removal, potentially affecting its PK/PD parameters [9, 40]. In these conditions, in line with Roger’s results, the severity of the condition may influence the drug disposition.

### Results for PK/PD parameters

Cmax and Cmin values are highly variable among patients considered in this review, as they ranged from 12.4 to 28.6 mg/L and from 1.7 to 14 mg/L, respectively (Table 1). Although Cmax values are similar or higher than those reported in the literature for healthy subjects, patients with renal impairment and ICU patients [13, 16], the Cmin values were lower than those reported for the same populations. Similarly, total clearance and volume of distribution values observed in the overall population were also highly variable (from 3.64 to 11.3 L/h and from 26.5 to 67.89 L, respectively) (Table 1). Patients’ comorbidities and organ dysfunction may significantly affect these parameters. For instance, the large Vd calculated by Roger et al. in the CVVH group might be explained by the likely greater fluid overload observed in these patients with respect to the CVVHDF group. Indeed, the net ultrafiltration fluid removal set in the CVVH group was about four times that in the CVVHDF group (net ultrafiltration 1100 vs 3620 ml respectively, see Table 1).

All these observations suggest that the clinical features of critically ill patients with sepsis and AKI treated with CRRT may play a key role in the achievement of appropriate PK/PD parameters for linezolid [36]. When considering pathogens with an MIC value of 2.0 mg/L for linezolid, two of the reviewed studies reported AUC/MIC ratios constantly below 85, and therefore not adequate for a clinical cure. What is more, when considering pathogens with an MIC value of 4.0 mg/L, only one study reported the optimal AUC/MIC ratio [15]. Therefore, the failure to achieve the optimal PK/PD targets for linezolid might increase the likelihood of emergence of microbial resistance and consequent clinical failure [36]. Furthermore, increased dosing of linezolid in critically ill patients with sepsis and AKI undergoing CRRT could expose patients to adverse effects. Thrombocytopenia has been reported as a frequent side effect of linezolid, especially in patients with renal dysfunction, in patients who develop heparin-induced thrombocytopenia (HIT) during anticoagulation treatment for CRRT, and in patients whose blood is exposed to low compatibility membranes.

Another partial explanation for our results may be related to linezolid drug-drug interactions. Drug-drug interaction with linezolid has been reported by several authors [14, 41–47] Rifampin can markedly decrease the linezolid serum concentration (thus affecting the probability of target attainment for clinical success). On the contrary, the majority of drugs interacting with linezolid, such as P-glycoprotein and CYP isoform inhibitors (i.e. protein pump inhibitors, calcium channel blockers, macrolides and so on), increase linezolid serum levels and therefore do not contribute to increased risk of clinical failure. Unfortunately, no information on pharmacological treatments is available for patients in the studies analyzed for this systematic review.

In this context, if optimal linezolid PK/PD targets are not achieved in patients with sepsis and AKI treated with CRRT, clinical failure of antibiotic treatment is more likely, especially in the case of infections sustained by microorganisms with higher MIC values (e.g. Enterococci). Therefore, although conflicting opinions are reported on the use of TDM for linezolid therapy monitoring in critically ill patients with sepsis, it is however, to be recommended for these patients, especially if they develop AKI and eventually CRRT is to be started [2].

## Conclusions

Wide variability in linezolid PK/PD parameters has been observed across critically ill patients with sepsis, especially those with AKI treated with CRRT. The effects of the extracorporeal treatment on antibiotic PK/PD target achievement should be carefully considered and adapted to the individual patient’s physio-pathological characteristics. Similar to other serious conditions, a TDM could be an effective method to ensure adequate antibiotic exposure, especially in critically ill patients with sepsis and AKI, who are on CRRT. If TDM is not routinely available, increased posology of linezolid might be alternatively considered for these patients during treatments performed with high diffusive/convective and/or adsorption clearance. Furthermore, different modalities of administration might be considered, such as continuous infusion.

## Abbreviations

AKI: acute kidney injury
APACHE II: Acute Physiology and Chronic Health Evaluation II
AUC_0–24_: area under the plasma concentration time curve
BW: body weight
CL_CRRT_: extracorporeal clearance
CLtot: total clearance
Cmax: antibiotic maximum serum concentration
Cmin: antibiotic trough
CRRT: continuous renal replacement therapy
CVVH: continuous veno-venous hemofiltration
CVVHD: continuous veno-venous hemodialysis
CVVHDF: continuous veno-venous hemodiafiltration
HCO: high cut-off membranes
ICU: intensive care unit
MIC: minimum inhibitory concentration
PK-PD: pharmacokinetic/pharmacodynamics
PRISMA: preferred reporting items for systematic reviews and meta-analyses
Qb: blood flow
Qd: dialysate flow
Qeff: effluent flow
Qf: replacement flow
Qf_NET_: net ultrafiltration flow
SA/SC: saturation coefficient or sieving coefficient
SOFA: Sequential Organ Failure Assessment
t_1/2_: plasma elimination half-life
TDM: therapeutic drug monitoring
UF_NET_: net ultrafiltrate
Vd: volume of distribution
X_CRRT_: total amount of drug eliminated by the extracorporeal treatment
